# Isolated Lymphatic Injury Mimicking Bladder Rupture in a Young Male Following Blunt Trauma: A Case Report

**DOI:** 10.1155/crra/4340538

**Published:** 2026-04-27

**Authors:** Prashant Kumar Gupta, Evan Botterman, Sara Morgan, Aarti Gupta

**Affiliations:** ^1^ Department of Radiology, Rutgers Health-Newark Beth Israel Medical Center, Newark, New Jersey, USA; ^2^ Rutgers University-New Jersey Medical School, Newark, New Jersey, USA, rutgers.edu; ^3^ Department of Internal Medicine, Rutgers Health-Newark Beth Israel Medical Center, Newark, New Jersey, USA

**Keywords:** bladder trauma mimicry, blunt abdominal trauma, chylous ascites, cisterna chyli, lymphatic injury, retroperitoneal injury

## Abstract

Blunt abdominal trauma can be difficult to evaluate, especially when an unusual injury mimics a far more common one. We present a case of a 17‐year‐old boy who was struck in the abdomen by bicycle handlebars. His initial imaging suggested a possible bladder rupture because of the presence of both intraperitoneal and extraperitoneal fluid. A retrograde CT cystogram, however, ruled out any bladder injury. Because of his worsening condition, he underwent an urgent laparoscopy followed by exploratory laparotomy, which revealed a large volume of milky‐white free fluid, suggestive of chyle, within the abdomen. Laboratory analysis of the aspirated fluid showed a high triglyceride level of 1,079 mg/dL, confirming the chylous nature of the fluid. There was an enlarged and engorged cisterna chyli in the retroperitoneum suggesting lymphatic injury. Treatment involved applying fibrin sealant in the region of the cisterna chyli, placing a peritoneal drain, and keeping a diet based on medium‐chain triglycerides. The patient recovered well without any recurrence of chylous ascites. This case highlights the need to consider rare lymphatic injuries causing posttraumatic chylous ascites when evaluating blunt abdominal trauma with diffuse abdominal fluid collection, particularly when common injuries have been ruled out but the patient′s clinical status continues to worsen.

## 1. Introduction

Blunt abdominal trauma (BAT) is a common reason for emergency department visits, most often caused by motor vehicle crashes, falls, or sports‐related injuries. Although damage to solid organs or the hollow viscera is easily recognized, less typical presentations can be much harder to identify [1, 2]. Bladder injury, one of the more common speculations in BAT, usually leads clinicians to obtain computed tomography (CT) cystography when it is suspected [3, 4]. Yet early imaging in BAT does not always give clear answers, and the findings can create a complicated diagnostic course [1, 2]. Lymphatic injury—especially when appearing as chylous ascites, is an exceptionally uncommon consequence of BAT and often presents with vague symptoms that may delay recognition [5, 6]. Because lymphatic injury is rarely considered in the trauma setting, where the nonspecific appearance of intra‐abdominal fluid on CT imaging is typically attributed to more common injuries such as bladder rupture or hollow viscus perforation, posttraumatic chylous ascites is frequently misdiagnosed. This case of a young male describes an unusual posttraumatic lymphatic injury that initially resembled bladder trauma after a blunt abdominal impact, emphasizing the diagnostic challenges and the importance of maintaining a broad differential in uncertain clinical presentations.

## 2. Case Presentation

A 17‐year‐old male with no significant past medical history presented to the emergency department 1 day after a bicycle accident where he struck his upper abdomen against the handlebars. Immediately following the injury, he experienced left‐sided abdominal pain, which had later shifted to the right side upon presentation to the hospital. He denied any associated symptoms of fever, nausea, vomiting, or diarrhea.

**Figure 1:**
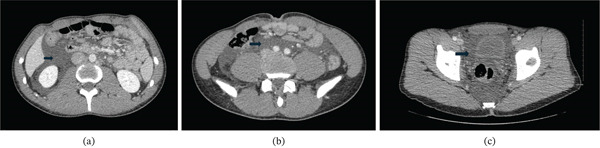
(a) Axial abdomen CECT image demonstrates free fluid in the perihepatic (intraperitoneal) and right perinephric (retroperitoneal) spaces. (b) Axial abdomen/pelvis CECT image demonstrates free fluid in the lower abdominal/upper pelvic cavity (intraperitoneal). (c) Axial pelvis CECT image demonstrates free fluid in the extraperitoneal pelvic cavity. Fluid collections are shown by arrows.

**Figure 2:**
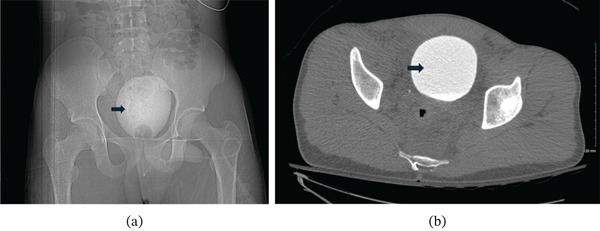
(a) CT scout view demonstrates contrast filled urinary bladder with its smooth and regular contour without extraluminal contrast material suggesting intact urinary bladder wall. (b) Axial pelvis image of retrograde CT urography suggests smooth contour of urinary bladder without evidence of bladder rupture. Arrows represent contrast filled urinary bladder.

During the presentation in the morning, the patient was hemodynamically stable, and his initial laboratory workup showed a normal complete blood count, normal liver and renal function tests. Within 1 h, a contrast enhanced CT scan of the abdomen and pelvis was done, which demonstrated free fluid in both intra and extraperitoneal cavities, with attenuation of approximately 15 Hounsfield units (HU). The fluid′s attenuation (HU) was measured with region of interest (ROI) methodology. The finding raised the suspicion for a combined bladder rupture (Figure 1). Further evaluation was subsequently done with a retrograde CT cystography (Figure 2). CT cystography was performed using retrograde filling of the bladder with approximately 300–400 mL of dilute water‐soluble iodinated contrast administered via a Foley catheter under gravity, ensuring adequate bladder distension prior to imaging. Imaging was obtained following passive filling without clamping. No extravasation of contrast was identified. The examination was performed in a 64‐slice GE Healthcare CT scanner with KVp of 120, mAs of 200, and slice thickness of 1.25 mm. The images were obtained without instillation of intravenous contrast.

This approach is consistent with current American College of Radiology (ACR) recommendations, which identify CT cystography as the diagnostic modality of choice when bladder injury is suspected. This subsequent examination, however, found no evidence of injury to the urinary bladder. Given the mechanism of injury, evaluation for thoracic injury was also considered. There was no clinical or radiographic evidence suggestive of blunt thoracic aortic injury or other thoracic pathology.

Shortly after imaging, and 8 h from initial presentation, the patient′s condition deteriorated acutely, and he became hemodynamically unstable with hypotension. Although his hemodynamic status showed transient improvement with resuscitative measures, persistent clinical deterioration and unclear imaging findings prompted emergent diagnostic laparoscopy to rapidly identify a potential intra‐abdominal source. Upon laparoscopy, a substantial volume of opaque, milky‐white fluid was immediately visualized within the abdominal and pelvic cavities. No hemoperitoneum or solid organ damage was identified. Upon inspection of the pelvis, there were no injuries or violations of the bladder. Milky fluid was visualized in the pelvis but not the source of the leak. During the laparoscopy, the right upper quadrant seemed to be the source of the leakage of fluid; however, despite suctioning, there was continuous reaccumulation of milky fluid in the area and absence of a visible definitive leak source. Therefore, the decision was made to proceed further with an exploratory laparotomy to enhance visualization, identify the definite source of fluid, and thoroughly assess for any bowel injury. The free fluid was also aspirated and sent for laboratory analysis, which confirmed a high triglyceride concentration of 1079 mg/dL, consistent with chyle. The creatinine analysis of fluid was not performed.

During the exploratory laparotomy, ongoing accumulation in the right upper quadrant of the abdomen was noted without evidence of bowel or solid organ injury. Retroperitoneal exploration was performed due to continued accumulation of milky fluid and concern for a possible occult retroperitoneal or lymphatic source of leakage. On opening the upper retroperitoneum, there was an engorged cisterna chyli with continuous leakage of milky fluid but no pinpoint source of lymphatic leakage. Therefore, Vistaseal fibrin sealant was applied to the retroperitoneum, and a 19‐French drainage catheter was placed within the retroperitoneum and peritoneum around the liver. After thorough abdominal irrigation with saline, the fascia and skin were closed.

Postoperatively, the patient was initially kept nil per os (NPO) and transitioned to a low‐fat medium‐chain triglyceride (MCT)‐based diet on postoperative Day 1 to reduce lymphatic flow and promote healing of lymphatic disruption. Continued supportive care and MCT‐based diet throughout the hospital stay led to significant clinical improvement during the hospital stay. He was discharged on postoperative Day 6 with the drainage catheter still in place to manage any residual collections and encouraged to remain on MCT‐based diet. At his 1‐week follow‐up, the drain output had decreased and was primarily serosanguinous, and the drain was maintained for continued monitoring. On the subsequent fifth‐week visit, he reported transient abdominal discomfort. Follow‐up imaging demonstrated a small partially loculated pelvic fluid collection, which was managed conservatively without the need for interventional radiology drainage. The drainage catheter was maintained to allow continued evacuation and monitoring of output. The drain remained in place for approximately 8 weeks, during which output progressively declined and ultimately ceased. At the time of drain removal, the patient remained asymptomatic. The patient returned to a regular diet by 8 weeks. He has continued outpatient follow‐up and remains clinically well, with no recurrence of chylous ascites or abdominal symptoms. The last follow‐up was done at four and a half months from the time of admission where he remained asymptomatic. A timeline summarizing the patient′s clinical course, diagnostic evaluation, interventions, and follow‐up is presented in Table 1.

**Table 1:** Timeline of clinical course.

Timepoint	Event
Day 0	Blunt abdominal trauma from bicycle handlebar impact
Day 1 (ED presentation)	Presented with abdominal pain; hemodynamically stable; initial laboratory evaluation unremarkable
Hour 1	Contrast‐enhanced CT abdomen/pelvis demonstrated intraperitoneal and extraperitoneal free fluid (~15 HU), raising suspicion for bladder injury
Hour 2–3	Retrograde CT cystography performed with adequate bladder distension; no contrast extravasation identified
Hour 8	Acute clinical deterioration with hypotension despite initial resuscitation
Day 1	Emergent diagnostic laparoscopy revealed large volume of milky intraperitoneal fluid without evidence of solid organ or bowel injury
Day 1	Conversion to exploratory laparotomy due to persistent fluid accumulation; retroperitoneal exploration revealed engorged cisterna chyli with suspected lymphatic leakage
Day 2 (postoperative Day 1)	Initiated low‐fat, medium‐chain triglyceride (MCT) diet
Day 7 (postoperative Day 6)	Discharged in stable condition with drain in place
Week 2 follow‐up	Decreased drain output; fluid serosanguinous in appearance
Week 6 follow‐up	Mild abdominal discomfort; imaging demonstrated small pelvic fluid collection managed conservatively
Week 9 follow‐up	Drain removed after cessation of output; resumed regular diet
4.5 Month follow up	Asymptomatic with no recurrence of chylous ascites or abdominal symptoms

## 3. Discussion

This case clearly demonstrates how difficult it can be to reach a correct diagnosis in BAT, especially when an unusual injury closely resembles a more familiar one [1, 2]. The patient arrived after a bicycle handlebar injury to the abdomen with abdominal pain and signs of hemodynamic compromise. His initial CT scan showed free fluid in both the intraperitoneal and extraperitoneal cavities, raising a reasonable suspicion for combined bladder rupture—an injury that, although not common, can carry substantial risk [3]. CT cystography, recommended by American College of Surgeons (ACS), American Urological Association (AUA), and ACR guidelines, remains the gold standard imaging modality for evaluating suspected bladder injury due to its high sensitivity and specificity [3]. In our case, however, the CT cystogram ruled out bladder injury, yet his worsening condition left little choice but to proceed with urgent surgical exploration.

Evaluating the lymphatic system adds another layer of complexity. Structures such as the cisterna chyli are challenging to assess because of their intricate anatomy and limited visibility on routine imaging [7]. The cisterna chyli, the abdominal origin of the thoracic duct, typically lies in the retrocrural region behind the aorta at roughly the T12–L2 levels and receives lymphatic drainage from the intestinal and lumbar trunks [7, 8]. Although often difficult to visualize, it may occasionally be seen on CT as an elongated or saccular structure measuring approximately 5 cm [7]. Thin section multidetector CT may help clarify its normal anatomical variations. These variations range from a narrow tubular structure to a round or oval configuration [7, 8]. When lymphatic injury is suspected, recognizing this structure becomes particularly important. CT may demonstrate fluid collections consistent with chylous ascites when present [6, 9]. However, identifying subtle lymphatic abnormalities requires careful radiologic evaluation. In our case, the engorged cisterna chyli was not visualized on CT, which contributed to delayed diagnosis.

During laparoscopy, the discovery of milky‐white fluid immediately shifted the diagnostic consideration away from bleeding or solid organ pathology. Identification of lymphatic injuries intraoperatively can be challenging because lymphatic vessels are small, fragile, and may not actively leak at the time of surgical evaluation. This diagnostic difficulty has been well described in the literature and contributes to delayed identification and management in many cases [10]. Further laboratory testing confirmed markedly elevated triglyceride levels in the aspirated fluid, establishing the diagnosis of chyle leakage and, therefore, a lymphatic injury [5]. Posttraumatic chylous ascites is exceedingly rare, with only a small number of documented cases in the literature [5, 6]. In this patient, the direct blow from the bicycle handlebars likely caused enough shearing or compressive force on retroperitoneal lymphatic structures, particularly the cisterna chyli, to cause an injury [11].

The diagnostic process was further complicated by the patient′s vague symptoms and the nonspecific initial CT findings. Free intraperitoneal fluid without a clear injured organ is a common but ambiguous feature in BAT [1]. The pattern of fluid initially raised the suspicion of bladder trauma, since its distribution could easily suggest urinary leakage. This case emphasizes that although advanced imaging is indispensable, persistent unexplained intraperitoneal or retroperitoneal fluid after a negative cystography, especially when the patient′s condition deteriorates, should prompt reevaluation of the differential diagnosis [4]. Such scenarios highlight why rare entities like posttraumatic chylous ascites often come into attention only after more common injuries have been systematically excluded [2].

From an imaging perspective, differentiating the cause of intra‐abdominal free fluid in trauma is critical. Intraperitoneal bladder rupture typically demonstrates contrast extravasation outlining bowel loops, whereas extraperitoneal rupture shows contrast confined to the perivesical space [12]. Urinomas generally appear as fluid collections on CT but are usually associated with identifiable urinary tract injury [13]. Bile leaks may demonstrate fluid collections with slightly higher attenuation and are typically associated with hepatobiliary injury [14]. Mesenteric injuries may present with fluid, bowel wall thickening, or mesenteric stranding [15].

In contrast, chylous fluid typically demonstrates low attenuation values (often < 20 HU) and may exhibit fat–fluid levels due to its lipid content. However, these findings are nonspecific and overlap with other etiologies of low‐density fluid. Therefore, biochemical analysis remains the reference standard for diagnosis, with triglyceride levels greater than 200 mg/dL strongly supporting chylous ascites. In this case, markedly elevated triglyceride levels confirmed the diagnosis of chylous ascites and helped distinguish lymphatic injury from other causes of intra‐abdominal fluid [16].

Management of traumatic chylous ascites generally starts with conservative therapy, like dietary modifications and image‐guided drainage [5]. In this case, however, surgical exploration was necessary due to the patient′s hemodynamic instability and the uncertain diagnosis [2]. Exploration allowed confirmation of the site of trauma with direct visualization of the lymphatic leak and placement of fibrin sealant to promote sealing of microscopic lymphatic leaks. Although evidence remains limited, fibrin sealant use in this case is supported by multiple reports demonstrating its efficacy in managing lymphatic injuries where direct ligation is not feasible [17–19]. A stepwise strategy including urgent surgery for stabilization and diagnosis followed by conservative lymphatic management proved effective for this patient, despite a brief period of increased abdominal pain and small fluid accumulation during recovery.

In cases of persistent or refractory lymphatic leakage, intranodal lymphangiography serves both a diagnostic and therapeutic role by identifying the site of lymphatic disruption and, in some cases, promoting spontaneous sealing [10]. Additionally, thoracic duct or cisterna chyli embolization has been increasingly utilized, with reported success rates exceeding 75% in select cases [10]. These interventions are generally considered when conservative management fails or when ongoing high‐output chyle leakage persists. In the present case, these techniques were not pursued due to the patient′s clinical stabilization following surgical intervention and successful resolution with conservative postoperative management with prolonged drainage and dietary modification without the need for interventional radiology or repeat surgical intervention [10].

Previously reported cases of traumatic chylous ascites are summarized in Table 2.

**Table 2:** Summary of previously reported cases of traumatic chylous ascites following blunt abdominal trauma.

Author (Ref)	Age	Mechanism of injury	Imaging findings	Diagnosis method	Management	Outcome
Fernandes et al. [5]	Adult	Blunt abdominal trauma	Free intraperitoneal fluid, no solid organ injury	Exploratory laparoscopy + peritoneal fluid triglyceride analysis (5142 mg/dL)	Conservative (low‐fat diet, long‐chain triglyceride restriction, drainage)	Resolved
Eren et al. [6]	Adult	Blunt abdominal trauma	Intraperitoneal and retroperitoneal fluid without solid organ injury	Diagnostic paracentesis with fluid triglyceride analysis (772 mg/dL)	Conservative (drainage, somatostatin, low‐fat diet with long chain triglyceride restriction)	Resolved
Calkins et al. [9]	Pediatric	Blunt trauma	Retroperitoneal fluid in the region of distal duodenum, no intraperitoneal fluid or solid organ injury	Exploratory laparotomy	Surgical repair of ruptured cisterna chyli	Resolved
Dissanaike et al. [11]	Adult	Blunt trauma	Fluid in the gallbladder fossa	Exploratory laparoscopy followed by laparotomy	Surgical repair of disrupted right lumbar branch entering the cisterna chyli	Resolved
Present Case	17 years old	Bicycle handlebar injury	Diffuse intraperitoneal and extraperitoneal fluid mimicking bladder rupture	Surgical + triglyceride (1079 mg/dl)	Fibrin sealant + MCT diet + drainage	Resolved

## 4. Conclusion

This case highlights that posttraumatic lymphatic injury presenting as chylous ascites should be considered in the differential diagnosis of BAT despite its rarity, especially in a case of diffuse abdominal fluid. This is particularly relevant when early imaging is ambiguous or does not fully match the patient′s clinical presentation, such as when bladder rupture is initially suspected. Though the diagnosis of lymphatic injury was not initially considered in this case, the positive outcome was obtained through early recognition of the patient′s clinical decline, timely surgical exploration, and confirmation of diagnosis and required management. Physicians should remember that unexplained abdominal fluid in a case of trauma may suggest uncommon injuries, and awareness of rare conditions along with multidisciplinary evaluation and coordination is critical for accurate diagnosis and proper management in complex trauma cases.

This report is limited by its single‐case design, which restricts generalizability. Additionally, lymphangiographic confirmation of the exact site of lymphatic injury was not performed, and diagnosis was based on intraoperative findings and biochemical analysis of aspirated fluid. Despite these limitations, this case provides valuable insight into the diagnostic challenges and management of traumatic lymphatic injuries presenting as chylous ascites.

## Funding

No funding was received for this manuscript.

## Ethics Statement

Ethical approval was not required for this case report.

## Consent

No written consent has been obtained from the patients, as there is no patient identifiable data included in this case report.

## Conflicts of Interest

The authors declare no conflicts of interest.

## Data Availability

The data that support the findings of this study are available from the corresponding author upon reasonable request.
